# Modulation of reward positivity and blink rate in children with attention deficit hyperactivity disorder (ADHD) during and following transcranial direct current stimulation

**DOI:** 10.1038/s41398-025-03720-w

**Published:** 2025-11-20

**Authors:** Jasper Vöckel, Lena Pokorny, Rebecca Rossberg, Nina Geist, Anne Kühnel, Nils B. Kroemer, Divya Seernani, Christoph Klein, Stephan Bender

**Affiliations:** 1 Department of Child and Adolescent Psychiatry, Psychosomatics and Psychotherapy, University of Cologne, Faculty of Medicine and University Hospital Cologne, Cologne, Germany; 2 Section of Medical Psychology, Department of Psychiatry and Psychotherapy, Faculty of Medicine, University of Bonn, Bonn, Germany; 3 Department of Psychiatry and Psychotherapy, Tübingen Center for Mental Health, University of Tübingen, Tübingen, Germany; 4 German Center for Mental Health (DZPG), Tübingen, Germany; 5 iMotions A/S, Copenhagen, Denmark; 6 Department of Child and Adolescent Psychiatry, Psychotherapy and Psychosomatics, University of Freiburg, Freiburg, Germany; 7 2nd Department of Psychiatry, National and Kapodistrian University of Athens, Medical School, University General Hospital “Attikon”, Athens, Greece

**Keywords:** Molecular neuroscience, Biomarkers

## Abstract

Transcranial direct current stimulation (tDCS) over the right pre-frontal cortex has been found to increase striatal dopaminergic activity and modulate dopaminergic dependent behavior. However, it remains to be elucidated to what extent this tDCS set-up is effective in changing reward system activation in children and adolescents with attention-deficit/hyperactivity disorder (ADHD). Twenty-four children and adolescents with ADHD received both, 2 mA and sham tDCS. The anode was positioned over the ventromedial prefrontal-cortex (PFC), while the cathode was placed over the right dorsolateral PFC. During and after tDCS, participants performed an effort allocation task aimed at earning monetary rewards. Concurrent with task performance, the reward positivity (RewP) and blink rate were assessed using electroencephalography and eye tracking. Both markers may be influenced by striatal dopamine levels. We found an increase in RewP after tDCS compared to sham (b = 0.65; p = 0.010), but no significant difference during stimulation. No significant differences were found for tDCS-related changes in blink rate during or after stimulation. tDCS with an anode over the vmPFC and cathode over the rDLPFC increases RewP after stimulation in children and adolescents with ADHD, which may be related to tDCS effects on reward processing.

## Introduction

ADHD (Attention-Deficit Hyperactivity Disorder) is the most common disorder in children and adolescents and is characterized by an increase in impulsivity, hyperactivity, and inattention [1, 2]. While the biological mechanisms of ADHD are heterogeneous, a common theory points to an underlying deficit in dopaminergic firing during the anticipation and feedback of rewards [3, 4]. These motivational deficits become evident in tasks requiring effort to achieve a rewarding outcome and are referred to as effort aversion [5, 6]. Dopamine-reuptake inhibitors (e.g. methylphenidate) effectively modulate such deficits on a behavioral and molecular level [5, 7–10]. However, their application is limited by side effects like a decrease in appetite [11], increased risk for cardiovascular diseases [12] or diminished growth [13]. Additionally, some subjects do not respond sufficiently to stimulant treatment [11]. Therefore, the development of targeted non-pharmacological treatments is needed. In this regard, transcranial direct current stimulation (tDCS) may be a promising treatment option. It can be applied at home [14] and is characterized by limited side effects, e.g. itching or mild burning under the electrode [15]. tDCS uses an anode and a cathode to modulate neuronal activity with a weak direct current [16]. Recently, a study placing the anode over the venteromedial prefrontal cortex (vmPFC) and the cathode over the right dorsolateral prefrontal cortex (rDLPFC) showed an increase of neuronal activity in the ventral striatum, which was similar to the neuronal activity seen after L-DOPA application [17]. However, neurobiological markers of dopaminergic changes during reward processing have not yet been investigated.

The *reward positivity* (RewP) is an event-related potential assessed through electroencephalography (EEG) with high temporal precision, commonly exhibiting a positive peak 200–400 milliseconds (ms) after reward presentation in behavioral paradigms [18]. Administration of the dopamine receptor antagonist haloperidol diminishes the amplitude of RewP [19] and the dopamine agonist d-amphetamine increases its amplitude [20]. Additionally, combining functional magnet-resonance-tomography (fMRI) and EEG analysis, a correlation of the hemodynamic activity of cortical reward processing regions (e.g. ventral striatum, mPFC) with the RewP was found [21, 22]. Consequently, the EEG-measured RewP may be strongly influenced by dopamine activity during reward processing, suggesting its potential as a biological marker in this domain.

A series of studies in animal models and human subjects has suggested a correlation between the *rate of eye blinks* and striatal dopaminergic activity [23]. Notably, dopamine agonists have been shown to increase the frequency of eye blinks [24, 25]. However, these pharmacological findings are limited to a very small number of included subjects in both studies.

Further, eye blink rate may be a marker of behavioral changes in reward processing. For example, an elevated rate of eye blinks was found during the anticipation and receipt of rewards, compared to baseline [26]. Moreover, in adolescents compared to adults, an increase in blink rate was observed during the reward maximization phase in a gambling task [27]. Lastly, in a button-pressing task aimed at earning monetary rewards, an increase in eye blink rate was observed from reward anticipation to reward receipt [28]. Therefore, eye blinking may be a promising marker to monitor neuronal changes in reward processing.

In this pre-registered study, children and adolescents with ADHD underwent tDCS stimulation, targeting the vmPFC with an anode and the rDLPFC with a cathode. Both during and after stimulation, participants engaged in an effort-demanding motor reward task. Within this task we manipulated several factors with changing difficulty levels and reward magnitude, which allowed subjects to decide how to maximize reward allocation and prevent exhaustion. In a previous publication, we reported an increase in effort maintenance, measured by the frequency of button presses, for delayed rewards with tDCS compared to sham in this sample [29]. Throughout the task, we measured the RewP and the rate of eye blinks in response to earned points which could later be exchanged for rewards. First, we hypothesized that, compared to sham stimulation, tDCS would result in an increased RewP, which would persist after stimulation. Second, we expected an increase in blink rate during and after tDCS compared to sham.

## Methods

### General procedure and participants

This study was a double-blind, sham-controlled, randomized, cross-over clinical trial and pre-registered (DRKS00029410). Blinding included subjects with ADHD, parents, and researchers during data acquisition. Informed consent was obtained from every child with ADHD and their parents. For randomization, a list of participant codes was generated with a changing block wise randomized order of treatment procedure. The trial included subjects with ADHD aged 8 to 17. Individuals with autism spectrum disorder, anxiety disorder (except specific phobia), tic disorder, personality disorder, depression, eating disorder, and an IQ below 85 were excluded. We included only subjects that did not take long-acting stimulant medication for 48 h and immediate-release stimulant medication for 24 h before the tDCS intervention.

Ethical approval was granted by the Ethics Committee of the Medical Faculty of the University Hospital of Cologne (ID: 00013774), the study adhered to the Declaration of Helsinki, the Good Clinical Practice Guidelines of the International Council for Harmonization [30] and European regulations for medical devices ((EU) 2017/745). Data were collected between November 2022 and April 2023.

**Fig. 1:**
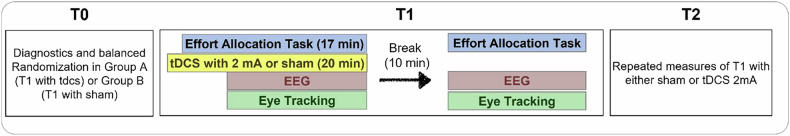
The study comprised three visits, including the completion of interviews and questionnaires (T0) and two stimulation sessions with either tDCS or sham (T1 and T2). Subjects were randomized to group A (tDCS at T1) or B (sham at T1). During and after stimulation, we recorded electroencephalography and used eye tracking to measure blink rate.

The study involved three site visits, incorporating diagnostic assessments and two visits for the intervention. Each participant underwent both tDCS and sham stimulation on separate days. In light of previous findings indicating that single-session tDCS does not produce lasting effects into the following day [31], we chose to implement a minimum interval of 24 h between stimulation conditions. The intervention consisted of 20 min (min) of tDCS/sham stimulation, with 3 min of stimulation alone and 17 min of stimulation during the effort allocation task, alongside EEG and eye tracking recordings. The effort allocation task, with concurrent EEG and eye tracking assessments, was repeated 10 min after completing tDCS/sham **(**Fig. 1). After each stimulation session, side effects were systematically assessed and blinding was evaluated by asking the participants about the stimulation modality.

### Sample

Screening procedure and diagnostic instruments are described in detail in the supplementary material. Of the 26 participants included into randomization, one participant was excluded due to a tic disorder, and one participant withdrew from the trial during sham stimulation, due to a lack of motivation to finish the effort task. The mean age of the remaining N = 24 subjects was 11.6 years (95% CI [10.7, 12.4]), with a mean IQ of 106 (95% CI [100.2, 112.7]). 17 subjects had a combined ADHD subtype, two had hyperactive/impulsive and five had inattentive subtypes. Additionally, subjects presented comorbid diagnoses: oppositional defiant disorder (n = 6), specific phobia (n = 4), disruptive mood dysregulation disorder (n = 2), insomnia (n = 6), diurnal enuresis (n = 1), and nocturnal enuresis (n = 1).

### Transcranial direct current stimulation

**Fig. 2:**
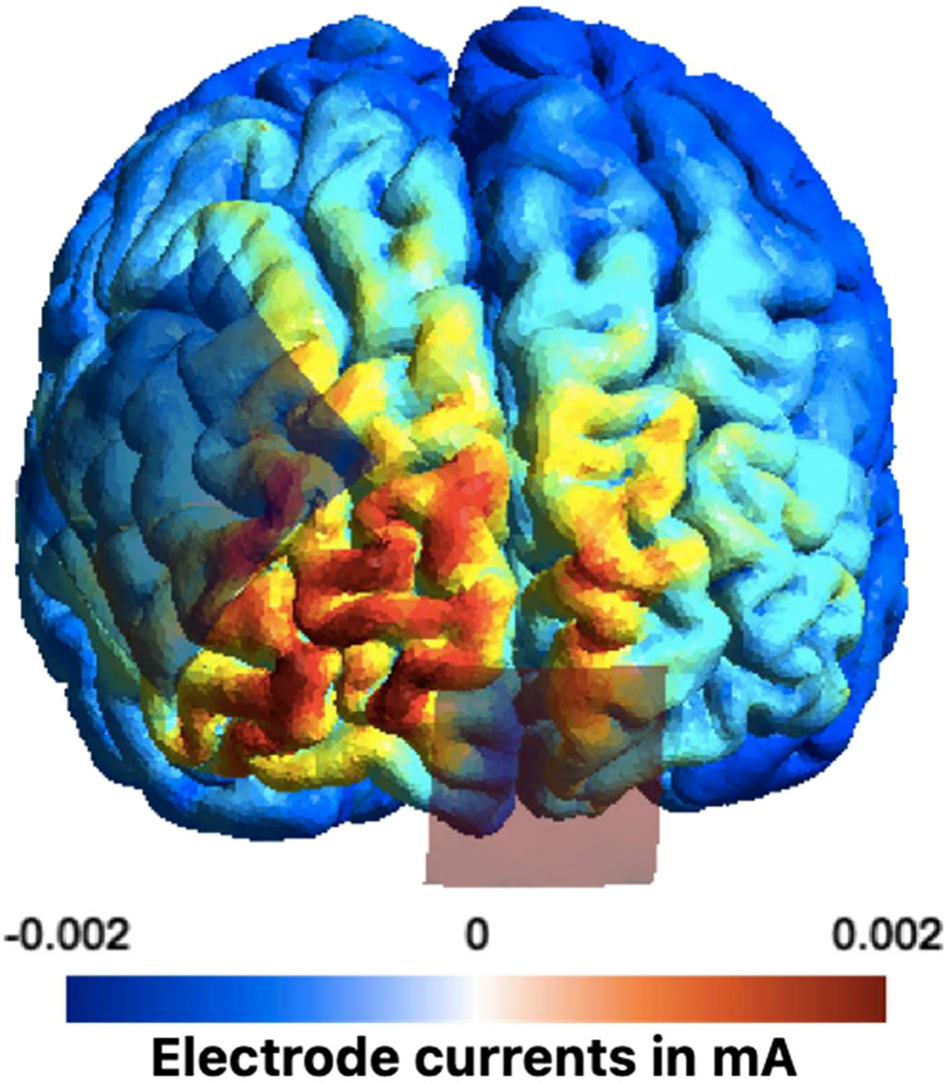
The anode was placed over the venteromedial pre-frontal cortex (Fpz) and the cathode over the right pre-frontal cortex (F4).

Each participant underwent stimulation using a battery-powered electrical stimulator (DC-Stimulator plus, NeuroConn, Ilmenau, Germany). The stimulation involved two conditions: active stimulation at 2 mA for 20 min (including a 30 s ramp-in and ramp-out) and sham stimulation on two separate days. The order of stimulation was both randomized and counterbalanced. The anode (3.5×3.5 cm) was positioned over Fpz, and the cathode (5×5 cm) was positioned over F4, using a 10–10- electrode placement system (Fig. 2[32],). Electrode impedance was kept below 5 kΩ at all times. Electrodes were placed with Ten20 conductive paste (Weaver and Company, Aurora, CO, USA). This specific electrode placement was chosen since it previously induced comparable neuronal activity as L-Dopa administration in mesostriatal regions in an fMRI study [17].

Sham stimulation involved a weak current of 110 µA delivered every 550 ms for 15 ms, producing side effects similar to tDCS.

### Effort allocation task

**Fig. 3:**
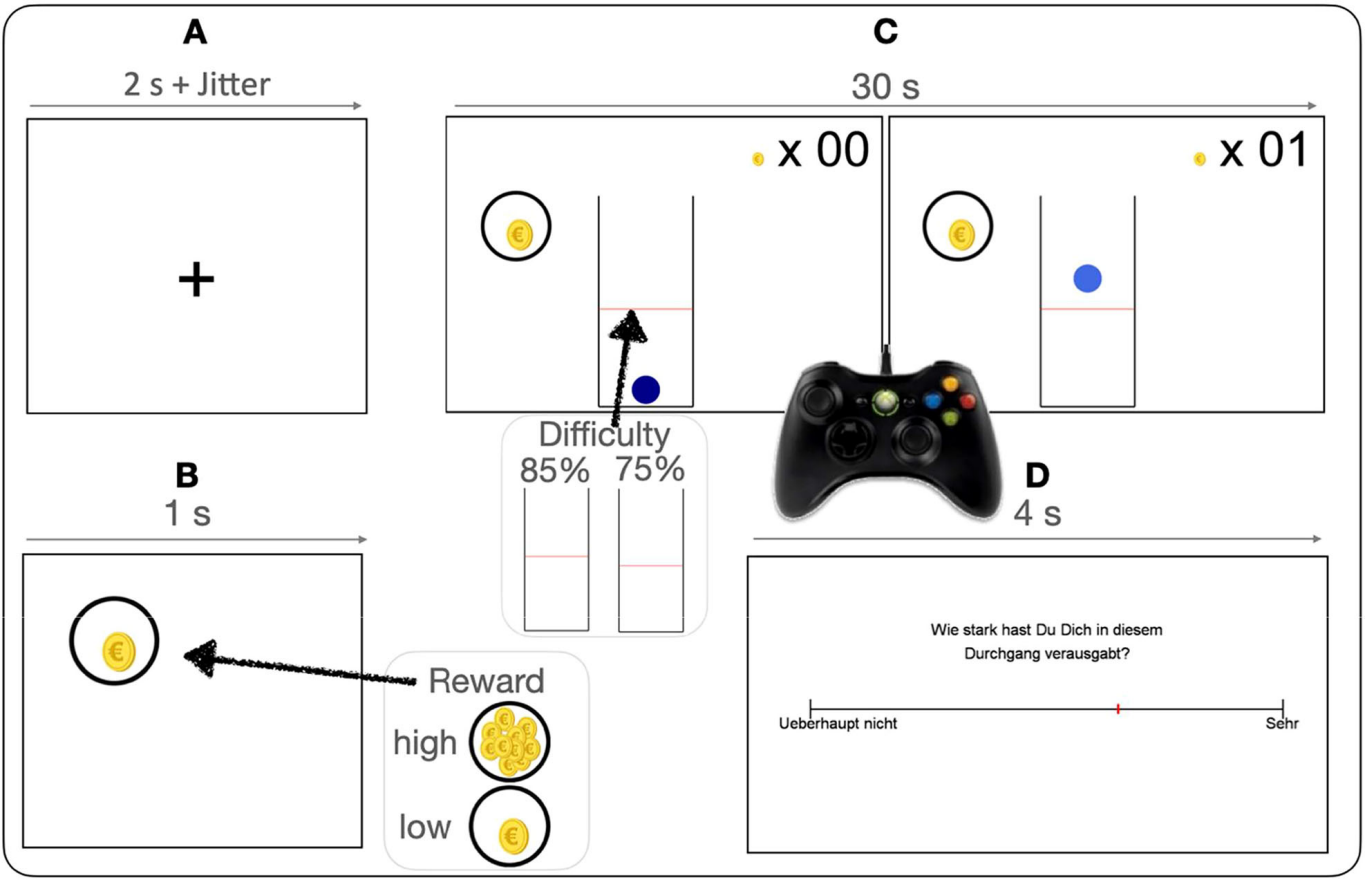
Exemplified trial procedure. First, a fixation cross was displayed for a duration of 2 s with varying jitter time intervals to prevent anticipation of the trial onset **A**. Next, a picture appeared for 1 s, indicating either the low or high reward condition in randomized order **B**. A display with a ball in a tube and a red line was presented, and subjects had to repeatedly press the button of an X-Box controller (Nacon, France) to keep the ball above the red line. For each second that the ball was held over the red line, subjects earned one point, and the total number of points earned in a trial was simultaneously displayed in a counter in the upper right corner of the screen. The level of difficulty (low vs. high) alternated **C**. After each trial, a visual analog scale was displayed to provide feedback on the level of exertion (**D**).

During stimulation and 10 min post-stimulation, participants engaged in a motor effort allocation task, each lasting 17 min **(**Fig. 3). This task comprised 24 trials, each lasting 30 s, with a 15 s rest period following the first 12 trials. The task was adapted from Neuser et al. [33]. Participants were instructed to repetitively press a button on an X-Box controller to maintain a ball presented on a computer screen above a red line, earning points for each successful second. EEG recording software received time-locked triggers every time a point was earned (i.e., when one second was completed). Trials varied in their reward magnitude (low: 1 point per s, high 10 points per s). Upon task completion, points were converted into monetary rewards. Specifically, participants had gained 5 cents for every 100 points accumulated. The task’s difficulty level varied by adjusting a red threshold line, which determined the position of the ball for earning points. The red threshold line alternated between low and high positions (low/high difficulty) in every trial. Upon task completion, participants viewed their earned points, and corresponding monetary amounts, and received the actual payout. Refer to Fig. 3 and its legend for a detailed trial procedure. The task was programmed using MATLAB and Psychophysics Toolbox extensions [34].

### EEG recordings

The EEG was continuously recorded from 16 channels at a sampling rate of 500 Hz with an online band-pass anti-aliasing filter from DC to 250 Hz using a BrainAmp DC amplifier (Brain Products GmbH, Munich, Germany). Following the 10–20 EEG system [32], we placed sintered Ag-AgCl electrodes (EasyCap, Germany) at Fz, FCz, Cz, Pz, Oz, F3, C3, P3, C4 and P4. The reference electrode was placed on the left mastoid and the ground electrode on POz. F4 was omitted due to the placement of the tDCS electrodes. Vertical and horizontal electro-oculogram were recorded from electrodes placed 1 cm above and below the left eye and bilateral to the outer canthi. All impedances were kept below 5 kΩ. Triggers were set automatically during the effort allocation task every time a point was won. Data preprocessing was conducted offline with the BrainVision Analyzer software (Brain Products, Gilching, Germany). EEG data was low-pass filtered (70 Hz) and notch filtered (50 Hz). Epochs were time-locked to the reward stimulus and had a length of 1300 milliseconds (ms), starting 500 ms before the trigger lasting until 800 ms thereafter. The first 200 milliseconds before each trigger served as a baseline. Artifacts were rejected automatically whenever a maximal allowed voltage step of 50 µV/ms was exceeded, a maximal allowed difference of values of 200 µV in 200 ms was found, or the activity was below 0.5 µV within an interval of 100 ms. After automatic artifact rejection data were again visually inspected. All EEG recordings were corrected for ocular artifacts using independent component analysis. Next, EEG recordings were corrected for linear DC drifts by linear regression analysis. To correct for artifacts (e.g., distortions of filtering procedures) produced by the short current applied during the sham condition, the time interval of 40 ms before and after the sham on/offset triggers was interpolated in sham trials. Such an interpolation was not necessary for the tDCS condition, as the constant current did not affect the EEG recordings. This was accomplished by applying a DC correction after the voltage shift induced by tDCS (before the baseline). RewP averages were calculated based on artifact-free segments from every participant. In the final step, we computed the grand average of the RewP at electrode site Fz from 150 ms to 350 ms following each earned reward. A task-based RewP (rather than a trial-based RewP) was necessary to have a sufficient number of trials to calculate the average. The average RewP of every subject and session was used to calculate the grand average by group (tDCS vs. sham). The time window aligns with the RewP interval and topography described in the literature [35]. We only included subjects with at least artifact free 30 segments in each session. This procedure has been proven sufficient to reliably analyze the RewP [36]. Three subjects (12 sessions) were completely excluded from our analysis because they earned only a very limited number of points during task performance, resulting in an insufficient number of trials to calculate the average RewP. Additionally, eight sessions from five different subjects were excluded because they did not meet the threshold of at least 30 successfully recorded segments after artifact correction.

### Eye tracking recording

**Fig. 4:**
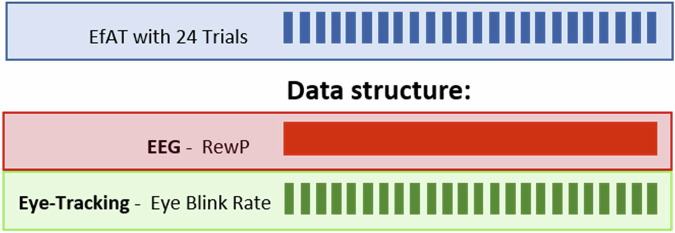
Description of the data structure. The EEG-RewP (time-locked to points won) was calculated based on every segment included during task performance across all 24 trials. The blink rate was based on trial-wise data output. EfAT = Effort Allocation Task, EEG=Electroencephalography.

Blinks were assessed with the Red250 mobile device (SensoMotoric Instruments, Germany) at a sampling rate of 60 Hz. Participants were seated 60–80 cm in front of the screen and calibration was performed at the beginning of each task. Blink data underwent preprocessing using Begaze (SensoMotoric Instruments, Germany) and were further analyzed using R. In line with SMI guidelines “blink” events shorter than 70 ms and longer than 1 s were discarded [34]. Additionally, we excluded trials with blinks that were three standard deviations above the mean of the group level. The output provided the blink count for each trial in every task. Consequently, 24 outputs at the trial level were generated for each task performance **(**Fig. 4). We only included trials with a tracking ratio above 60% (proportion of recorded time during a trial), which led to the exclusion of 28.64% of trials.

### Statistics

#### Pre-registered hypothesis

*Blink-Rate Analysis:* We analyzed changes in blink rate during task performance both concurrently with and after tDCS compared to sham stimulation, using two separate multilevel mixed-effects model. This model was appropriate given the hierarchical structure of the data. While for RewP analysis, individual averages were analyzed, in agreement with previous literature, for blink rate we analyzed single trials (trials 1–24) nested within each subject. To estimate the dependent variable, blink rate, our model included treatment (tDCS vs. sham) as the primary independent variable, along with the predictors difficulty (high vs. low), reward magnitude (high vs. low), age, sex, and session sequence. The model included random intercepts for each individual and employed maximum likelihood estimation. Using a per-family Bonferroni correction approach we adjusted the alpha level to.025 for the two blink rate analyses (i.e., during and after stimulation).

*RewP Analysis:* We employed a Generalized Least Squares (GLS) regression model to analyze our EEG data, accommodating both random (random intercepts for each individual) and fixed effects. A GLS model was used due to the non-hierarchical panel data structure with an averaged individual reward positivity based on every task performance. For both, task performance concurrent and after, the dependent variable, RewP, was subjected to a model including treatment (tDCS vs. sham), session sequence, the number of segments where points were won and RewP was successfully recorded, age, sex and stimulations sequence (to control for potential carry-over effects) as predictors. Notably, compared to our blink analysis, the predictors reward magnitude and task difficulty were not included, as these were trial-based and thus not applicable to the task-based averaged analysis of RewP. Again, we employed a Bonferroni correction with an adjusted alpha level at 025.

## Results

### Reward positivity (RewP)

**Fig. 5:**
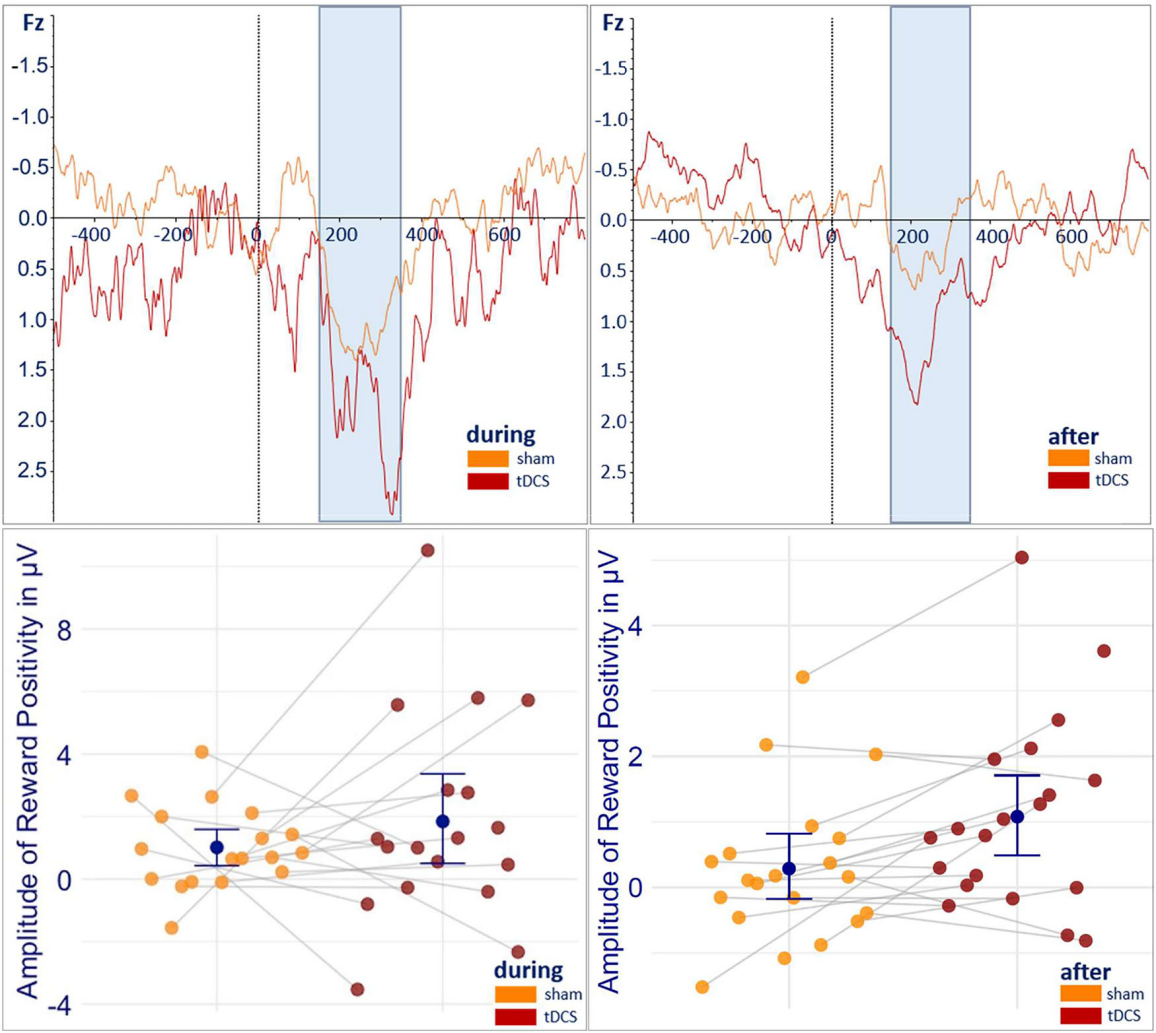
Amplitude of the Reward Positivity. Top Grand average over Fz of the amplitude of the reward positivity during task performance concurrent with and after tDCS vs. sham. Bottom: Individual data points and mean of the amplitude of the reward positivity (95% confidence interval) concurrent (left) and after (right) stimulation.

There was no significant difference in RewP amplitude during task performance concurrent with tDCS compared to sham (b = 0.76; z(12) = 0.79; 95% CI [−1.13, 2.67]; p = 0.43). However, after stimulation, we observed a significant increase in RewP amplitude during the task in the tDCS condition compared to sham (b = 0.65; z(14) = 2.58; 95% CI [0.16, 1.15]; p = 0.010, Fig. 5). For the analysis of the condition “task performance during stimulation” (Chi^2^ = 5.26; p = 0.511; R2 = 0.13) we included 36 sessions of 19 subjects, with 18 subjects having assessments for both tDCS and sham. For the analysis of the condition “task performance after stimulation” (Chi^2^ = 16.79; p = 0.010; R2 = 0.31) we analyzed 40 sessions of 21 subjects, with 19 subjects included in both stimulation settings. By including random effects in our model, we were able to accommodate all assessments, even those with only one time point. However, restricting the analysis to subjects with complete assessments proved that neither the inclusion or exclusion of subjects with incomplete datasets introduced a bias into our results (during: mean difference(md) = 0.77, t(16) = 0.41, p = 0.41; after: md = 0.58, t(18) = 2.76, p = 0.01; see supplementary material). Additionally, we found that younger subjects exhibited a higher RewP amplitude during (b = -0.37; z(12)= −1.29; 95% CI [-0.94, 0.19]; p = 0.195) and after(b = -0.32; z(14)= −2.14; 95% CI [-0.62, -0.02]; p = 0.033) stimulation, which only reached significance post-stimulation. None of the other predictors reached statistical significance in either model (see supplementary material).

### Blink rate analysis

We found no significant difference in blink rate during tDCS (b = 0.46; t(831.29) = 1.42; 95% CI [-0.17, 1.08]; p = 0.155; Table 1) compared to sham with a model including 852 trials of 24 subjects (F_6111.48_ = 5.12; p < 0.001). However, in the same model we found an increase of blink rate during trials with high difficulty compared to a low difficulty level (b = 0.85; t(824.65) = 2.77; 95% CI [0.25, 1.45]; p = 0.006). Additionally, we found a significant decrease with increasing age (b = −1.29; t(20.84)= −3.14; 95% CI [−2.15, -0.44]; p = 0.005) and a significant increase with increasing session sequence (b = 0.44; t(20.37) = 2.71; 95% CI [0.12, 0.76]; p = 0.007). Neither reward magnitude, nor sex significantly changed blink rate.

**Table 1:** Effect of tDCS (2 mA vs Sham) on blinks during overall task performance. We did not observe a significant difference of blinks concurrent with or after stimulation.

Blink Rate	Coefficient	t-Value	95% conf. interval	p-Value
Concurrent with stimulation	0.46	1.42	[-0.17, 1.08]	0.155
After stimulation	-0.02	−0.06	[-0.72, 0.67]	0.954

In the model examining blink rate after stimulation, we included 775 trials of 23 subjects (F_6102.48_ = 2.88; p = 0.012). We found no significant difference in blink rate during task performance after stimulation (b = -0.02; t(748.33)= -0.06; 95% CI [0.72, 0.67]; p = 0.954; Table 1). Blink rate decreased with increasing age (b = −1.17; t(19.19)= −2.50; 95% CI [−2.03, -0.18]; p = 0.022). Interestingly, we found a decrease in blink rate in trials with high reward magnitudes compared to trials with a low reward magnitude (b = −1.12; t(751.14)= −3.15; 95% CI [−1.83, -0.43]; p = 0.002). None of the other covariates (level of difficulty, session, sex) reached significance in the model (supplementary material).

## Discussion

The study aimed to elucidate potential reward-related neurobiological changes induced by tDCS with an anode over the vmPFC and a cathode over the rDLPFC in children and adolescents with ADHD during an effort paradigm. The EEG-RewP and eye blink rate were examined during tDCS with 2 mA compared to sham stimulation. We found an increase in the amplitude of the RewP during task performance after tDCS compared to sham. We did not observe a significant change in overall blink rate either during task performance concurrently or after stimulation.

This study found an increase in the amplitude of the RewP in our effort reward task performed after tDCS with 2 mA compared to sham. Previously, reduced amplitude of the RewP and EEG - feedback negativity (or feedback error negativity) has been reported in children and adolescents with ADHD compared to healthy controls [37–41]. Although two studies found no difference between youth with ADHD and healthy controls [42, 43], the majority of evidence suggests a deficit in reward feedback processing in ADHD, reflected here in the underlying neuronal activity of the RewP (or feedback negativity). As this study found an increase in RewP during task performance after tDCS, this finding may indicate that our tDCS setting could ameliorate the deficit in feedback reward processing by altering the RewP in youth with ADHD.

No significant change in the RewP was observed during task performance concurrent with stimulation, suggesting that our findings may be specific to the subsequent (longer-lasting) effects of tDCS. Visually, however, a higher fluctuating RewP was noted during compared to following stimulation across both stimulation conditions. In line with this visual observation, we observed a statistical significant reduction in successfully recorded RewP trials with concurrent stimulation compared to after stimulation, which may be due to artifacts induced by the constant current during tDCS with 2 mA, and the changing current during sham stimulation. Moreover, while the number of included EEG RewP trials after stimulation was comparable between the tDCS with 2 mA and sham condition, we observed a reduction in the number of included RewP trials during tDCS with 2 mA compared to sham (supplementary material) concurrent to stimulation. This points to a reduction in EEG data quality during stimulation, which limits the interpretation of the non-significant difference observed during stimulation and highlights the need for larger sample sizes to evaluate EEG event related potentials recorded concurrent with stimulation versus those recorded following it. Therefore, the limited sample size may have reduced our ability to detect a tDCS effect on reward processing during ongoing stimulation.

In our study, we used a sham procedure with a 30 s ramp-up at the beginning and a 30 s ramp-down at the end of stimulation at 2 mA. In between, every 550 ms, a current pulse of 110 μA for 15 ms (maximum current for about 3 ms) was delivered. This pulse is used for impedance checks and is assumed to have minimal neurobiological effects. As we did not include a no-tDCS control condition, we cannot exclude potential effects of the sham stimulation, which may have attenuated the reported effect sizes of 2 mA tDCS. In previous studies, sham tDCS—compared to no tDCS—resulted in performance improvements in an orientation discrimination task [44]. Therefore, sham effects may have been due to placebo responses, potentially triggered by side effects and participants’ expectations of an effect. Sham stimulation may also be associated with neurobiological effects on electroencephalographic parameters due to the small current applied [45]. For example, one study reported an increase in the EEG event-related potential P3 during a working memory task that was similar across sham, 1 mA, and 2 mA tDCS, compared to no tDCS [46]. Future study designs could address this by including a no-tDCS condition or by using topical pretreatments to minimize biological and placebo effects of tDCS. However, the use of topical pretreatments is limited in children and adolescents due to ethical considerations.

We did not observe differences in blink rate between sham and tDCS both concurrent with and after stimulation. The finding is in line with a previous study using tDCS with an anode over the rDLPFC and cathode above the lDLPFC – a set-up which has also shown to increase neuronal dopaminergic activity following tDCS [47], but did not induce differences in blink rate between tDCS and sham [48]. It is possible that overall blink rate is not a reliable marker for measuring neuronal dopamine-dependent changes. This assumption is supported by findings from studies using in vivo positron emission tomography, which found no correlation with blink rate to dopamine synthesis capacity und dopamine receptor availability [49–51]. In addition, the administration of dopamine agonists in subjects with depression [52] and ADHD [53] did not results in changes in blink rate.

However, the results of this study may also be influenced by specifics of the task design, which could have affected the sensitivity of blink rate as a marker of dopaminergic changes in reward processing. The task included various conditions, such as changing levels of difficulty (high vs. low) and reward magnitude (high vs. low), all of which could have affected the blink rate and may have masked specific changes in the blink rate by tDCS. In line with this hypothesis, we did observe an increase in blink rate during trials with a higher level of difficulty during stimulation, a finding, which is supported by a previous study, which showed higher levels of difficulty to be related to an increased eye blink rate [54]. However, during task performance after stimulation we were not able to replicate this finding, pointing to an inconsistency or a specificity to task performance concurrent with tDCS. Additionally, we observed a decrease in blink rate after stimulation during trials with high rewards, which may be explained by overlapping cognitive processes within our task design (again no consistency during and after stimulation). For example blink rate has been shown to decrease with increasing attentional demands [55]. Additionally, in a working memory task including rewarding and non-rewarding blocks, a decrease in blink rate for rewarding blocks was found, which may have been related to an increase in cognitive control [56]. This finding is in line with another study, showing less blinks in participants when more relevant content is viewed [57]. Therefore, our findings (no difference between tDCS and sham stimulation, and inconsistencies within difficulty and reward levels) may be attributed to changing overlapping processes (e.g. reward processing, cognitive control, attentional demands) as well as stimulation effects.

Whether tDCS results in changes of neuronal activity concurrent or after stimulation of the cortical reward network remains a matter of debate. Increased dopaminergic activity has been reported directly after or within a delay of 20–30 min after stimulation over the prefrontal cortex using positron emission tomography [47] and fMRI [17]. Conversely, magnetic resonance spectroscopy during and immediately after prefrontal tDCS found an increase in striatal neuronal glutamate + glutamine activity during, but not after stimulation [58]. The findings of this paper may support stronger increases in neuronal activity after and not during tDCS compared with sham. However, in the same sample, a behavioral increase in effort maintenance was found both during and after stimulation [29], which supports the assumption of both an immediate and delayed behavioral effect induced by tDCS. One might speculate that during tDCS, neuronal changes are more unspecific and therefore more difficult to detect, while effects on the reward network are more pronounced after stimulation. However, this hypothesis needs to be further elucidated by follow-up studies.

The results of this study need to be interpreted with several limitations. Both blink rate and RewP changes were pre-registered hypothesis, however as secondary outcome parameters they were not included in a pre-registered power analysis. A small number of subjects were included and results need proper replication in a larger sample. We only measured changes in neuronal activity of the RewP or blink rate in response to rewards. While changes underlying these read-outs may be dopamine-dependent, effects of other neuromodulators (e.g. noradrenaline, serotonin) cannot be excluded. Additionally, for our eye-tracking data, we allowed a data loss of 40% per trial. This was done, to overcome a bias in selecting only subjects with a very high tracking ratio. Lower quality in eye tracking data in younger subjects (especially with increasing trial sequence) is not unusual and has been shown before [59, 60]. This study did face the additional challenge of subjects with ADHD being off-stimulants and performing a task involving motor movements. To assure the validity of our data we performed an analysis correlating blink rate at 60% data availability with blink rate at 80% tracking ratio. This analysis confirmed a good inter-validity (60% tracking ratio: r = 0.67, p <0.001; 80% tracking ratio: r = 0.61, p < 0.001) and intra-validity (60% vs. 80% tracking ratio: b = 0.98, p < 0.001). Additionally, there was no significant difference in tracking ratio between tDCS with 2 mA vs. sham both during and after stimulation.

## Conclusion

In conclusion, we observed an increase in RewP amplitude, potentially indicating an increase in dopaminergic feedback during reward receipt following tDCS compared to sham in children and adolescents with ADHD, with the anode placed over the vmPFC and the cathode over the rDLPFC. Methodologically, future studies should consider larger sample sizes to detect concurrent effects of stimulation, as these may require greater samples than those needed for effects recorded post-stimulation. Additionally, the blink rate may need to be assessed in task designs with reduced confounding factors (e.g., difficulty, reward magnitude) to serve as a sufficiently sensitive marker.

## Supplementary information

Supplemental Material

## Data Availability

Anonymized data may be made available from the corresponding author upon reasonable request and subject to approval by the appropriate institutional ethics committee.
