# Supplementary material for: Modulation of reward positivity and blink rate in children with attention deficit hyperactivity disorder (ADHD) during and following transcranial direct current stimulation
Source: Transl Psychiatry. 2025 Nov 20;15:513. doi: 10.1038/s41398-025-03720-w (PMC12669705; doi:10.1038/s41398-025-03720-w)
Supplement: Supplementary file 1 — Supplemental Material [file 41398_2025_3720_MOESM1_ESM.docx]

**Supplementary Material**

**Modulation of reward positivity and blink rate in subjects with attention deficit hyperactivity disorder (ADHD) during and following transcranial direct current stimulation**

Jasper Vöckel^1^, Lena Pokorny^1^, Rebecca Rossberg^1^, Nina Geist^1^, Anne Kühnel^2^, Nils B Kroemer^2,3,4^, Divya Seernani^5^, Christoph Klein^1,6,7^, Stephan Bender^1^

^1^ Department of Child and Adolescent Psychiatry, Psychosomatics and Psychotherapy, University of Cologne, Faculty of Medicine and University Hospital Cologne, Cologne, Germany

^2^ Section of Medical Psychology, Department of Psychiatry and Psychotherapy, Faculty of Medicine, University of Bonn, Bonn, Germany

^3^ Department of Psychiatry and Psychotherapy, Tübingen Center for Mental Health, University of Tübingen, Tübingen, Germany

^4^ German Center for Mental Health (DZPG)

^5^ iMotions A/S, Copenhagen, Denmark

^6^ Department of Child and Adolescent Psychiatry, Psychotherapy and Psychosomatics, University of Freiburg, Hauptstr. 8, 79104 Freiburg, Germany

^7^ 2^nd^ Department of Psychiatry, National and Kapodistrian University of Athens, Medical School, University General Hospital "Attikon", Athens, Greece

1. **Screening procedure**

Upon obtaining informed written consent from each child and their legal guardians, a screening process was conducted. The diagnosis of ADHD, following the DSM-5 guidelines, was confirmed and categorized through a clinical interview administered by a child and adolescent psychiatrist using the Kinder-DIPS (57). Additionally, the severity of ADHD symptoms underwent evaluation using the DISYPS-III ADHD self- and parent rating scales (58). Right-handedness was determined using the Edinburgh Handedness Inventory (59). For cognitive assessment, the Culture Fair Intelligence-20 Revised Test was conducted for 17 out of 24 participants, with the remaining participants having undergone a valid IQ assessment within the preceding two years (60). In instances where prior IQ measurements were available, assessment relied on either the Wechsler Intelligence Scale for Children (n=6, (61)) or the Kaufman Assessment Battery for Children (n=1, (62)).

1. **RewP- GLS Model: Task performance during stimulation**

|  |  |  |  |  |  |  |
| --- | --- | --- | --- | --- | --- | --- |
|  | Coefficient | std. err. | z | P-value | [95% conf. interval] | |
| tDCS vs. sham | .7669847 | .9723715 | 0.79 | 0.430 | -1.138828 | 2.672798 |
| number of trials | -.0018425 | .0056954 | -0.32 | 0.746 | -.0130052 | .0093203 |
| Session sequence | .0237906 | .3901423 | 0.06 | 0.951 | -.7408743 | .7884555 |
| age | -.3756286 | .2901227 | -1.29 | 0.195 | -.9442587 | .1930014 |
| sex | -.8140871 | .5911967 | -1.38 | 0.169 | -1.972811 | .3446372 |
| Stimulation sequence | -.7965299 | .9338319 | -0.85 | 0.394 | -2.626807 | 1.033747 |
| _cons | 6.347417 | 3.200419 | 1.98 | 0.047 | .0747099 | 12.62012 |

1. **RewP-GLS Model: Task performance after stimulation**

|  |  |  |  |  |  |  |
| --- | --- | --- | --- | --- | --- | --- |
|  | Coefficient | std. err. | z | P-value | [95% conf. interval] | |
| tDCS vs. sham | .6517546 | .2525682 | 2.58 | 0.010 | .1567301 | 1.146779 |
| number of trials | -.0001326 | .0027499 | -0.05 | 0.962 | -.0055223 | .0052571 |
| Session sequence | .0720217 | .1339658 | 0.54 | 0.591 | -.1905465 | .3345899 |
| age | -.3239608 | .1515939 | -2.14 | 0.033 | -.6210794 | -.0268422 |
| sex | -.1670479 | .4211755 | -0.40 | 0.692 | -.9925368 | .6584409 |
| Stimulation sequence | -.5091011 | .5702881 | -0.89 | 0.372 | -1.626845 | .6086431 |
| _cons | 4.322713 | 1.925472 | 2.25 | 0.025 | .5488562 | 8.096569 |

1. **EEG-Analysis only with subjects with assessments both during tDCS with 2 mA and sham**

In our analysis we used a generalized least squares (GLS) model with random and fixed effects, accounting for both between and within subject variability. Using this approach in our analysis, we were able to include all EEG sessions, even when some subjects had incomplete assessments (e.g., missing assessments during sham or 2 mA tDCS) (63). However, the inclusion of subjects with some missing measures might have introduced bias into our data. To address this concern, we conducted an additional analysis using a t-test to compare the amplitude of the RewP during and after stimulation. In this analysis, we included only those subjects with a minimum of 30 successfully recorded RewP segments and complete data. For instance, in the t-test comparing post-stimulation performance, we included only subjects with successfully recorded data after both sham and 2 mA tDCS sessions.

Consistent with our analysis using the GLS model, we found a non-significant difference in the amplitude of the RewP during task performance with stimulation (mean difference [MD] = 0.77; t(16) = 0.86; 95% CI [-1.14, 2.69]; p = 0.403; Figure 1) and a significantly increased amplitude of the RewP post-stimulation after tDCS compared to sham (MD = 0.58; t(18) = 2.76; 95% CI [0.14, 1.04]; p = 0.012; Figure 2).

Figure 1: Whisker plot showing the difference in Reward Positivity between 2 mA tDCS and sham stimulation during task performance concurrent with stimulation.

Figure 2: Whisker plot showing the difference in Reward Positivity between 2 mA tDCS and sham stimulation during task performance after stimulation. We found a significant increase of the amplitude of the RewP during task performance after tDCS vs sham.

1. **Difference in included trials for EEG-RewP assessments**
   1. **Comparison of assessments tDCS vs. sham**

We found a significantly reduced number of recorded EEG-RewP trials (b=-56.66; z= -3.15; 95% CI [-91.95; -21.37]; p=.002) during stimulation with 2 mA (mean: 115.8; 95% CI [73.52; 158.25]) vs. sham (mean: 169; 95% CI [124.2; 213.78]). We observed a similar number of recorded trials after stimulation (b=-10.46; z= -.60; 95% CI [-44.61; 23.68]; p=.548) with a mean of 180.9 (95% CI [143.32; 218.48]) trials following tDCS with 2 mA and a mean of 188.75 (95% CI [145.99; 231.51]) following sham stimulation.

- 1. **Comparison of assessments during vs. after stimulation**

We found a significantly increased number of recorded EEG-RewP trials ( b=51.76; z= 7.08; 95% CI [37.42; 66.09]; p<.000) following stimulation (mean: 184.83; 95% CI [157.64; 212.01]) vs. concurrent with stimulation (mean: 142.44; 95% CI [111.83; 173.06]).

1. **Blink rate analysis**
   1. **Blink rate during stimulation (allowing 60 % tracking ratio)**

|  | b | Std. error | t-value | 95%-CI | p-value |
| --- | --- | --- | --- | --- | --- |
| Stimulation (tDCS vs. sham) | .46 | .32 | 1.42 | [-.17; 1.09] | .155 |
| Reward magnitude (high vs. low) | .46 | .31 | 1.51 | [-.14; 1.06] | .132 |
| Difficulty (high vs. low) | .84 | .31 | 2.77 | [.25; 1.45] | **.006** |
| Session sqeuence | .43 | .16 | 2.67 | [.12; .75] | **.008** |
| Age | -1.29 | .41 | -3.14 | [-2.15; -.43] | **0.005** |
| Sex | -.74 | 2.4 | -0.31 | [-5.74; 4.26] | .762 |

- 1. **Blink rate after stimulation (allowing 60 % tracking ratio)**

|  | b | Std. error | t-value | 95%-CI | p-value |
| --- | --- | --- | --- | --- | --- |
| Stimulation (tDCS vs. sham) | -.019 | .35 | -.05 | [-.72; .67] | .957 |
| Reward magnitude (high vs. low) | -1.12 | .36 | -3.15 | [-1.83; -.42] | **.002** |
| Difficulty (high vs. low) | .31 | .36 | .84 | [-.41; 1.01] | .401 |
| Session sqeuence | -.09 | .18 | -0.53 | [-.45; .26] | .599 |
| Age | -1.11 | .44 | -2.5 | [-2.03; -.18] | .022 |
| Sex | -1.17 | 2.47 | -0.47 | [-6.35; 4.01] | .641 |

- 1. **Blink rate during stimulation (allowing 80 % tracking ratio)**

|  | b | Std. error | t-value | 95%-CI | p-value |
| --- | --- | --- | --- | --- | --- |
| Stimulation (tDCS vs. sham) | .08 | .34 | 0.24 | [-.58; .75] | .809 |
| Reward magnitude (high vs. low) | .46 | .32 | 1.42 | [-.17; 1.09] | .155 |
| Difficulty (high vs. low) | .56 | .32 | 1.72 | [-.07; 1.19] | .086 |
| Session sqeuence | .38 | .17 | 2.25 | [.05; .72] | **.025** |
| Age | -1.21 | .48 | -3.08 | [-2.52; -.48] | **0.006** |
| Sex | -1.21 | 2.8 | -0.43 | [-7.03; 4.23] | .67 |

- 1. **Blink rate after stimulation (allowing 60 % tracking ratio)**

|  | b | Std. error | t-value | 95%-CI | p-value |
| --- | --- | --- | --- | --- | --- |
| Stimulation (tDCS vs. sham) | -.04 | .42 | -.1 | [-.88; .79] | .917 |
| Reward magnitude (high vs. low) | -.89 | .44 | -2.06 | [-1.75; -.42] | **.040** |
| Difficulty (high vs. low) | .22 | .44 | .52 | [-.63; 1.08] | .602 |
| Session sequence | .51 | .21 | 0.52 | [.08; .93] | **.020** |
| Age | -1.09 | .44 | -2.45 | [-2.03; -.15] | **.026** |
| Sex | -1.04 | 2.44 | -.43 | [-6.21; 4.13] | .675 |

1. **Difference in missing data of tDCS with 2 mA vs. sham in eye-tracking**
   1. **Missing data during task performance concurrent with stimulation**

We found no significant difference in missing data between tDCS vs. sham in our eye tracking recoding concurrent with stimulation (b = 6.51; t = .46; 95% CI [-20.99, 34.02]; p = .642). During tDCS we found a mean of 369.47 (95% CI [341.61, 397.33]) and during sham a mean of 365.14 (95% CI [340.01, 390.27]) missing data, corresponding to a mean of 6 seconds of missing data per trial in the sample.

- 1. **Missing data during task performance concurrent with stimulation**

Again, during task performance after tDCS vs. sham we found no significant difference in missing data (b = -1.6; t = .00; 95% CI [-28.24, 28.24]; p = 1). During tDCS we found a mean of 785.39 (95% CI [731.40, 839.37]) and during sham a mean of 785.39 (95% CI [731.41, 839.37]) missing data, corresponding to a mean of 12 seconds of missing data per trial in the sample.

1. **Validity of blink data**
   1. **Internal Validity**

Limitations in the interpretations of the blink data arise from the allowed tracking ratio of at least 60%. Using a higher tracking ratio leads to a loss of data (subjects, trials), which may not be appropriate for generating sufficient statistical models. To ensure internal validation of blinks, we correlated the dependent variable (blinks) at even and odd trials at a 60% tracking ratio (r = .67, p < .001) and an 80% tracking ratio (r = .61, p < .001). These results indicate good internal validity.

- 1. **Intra-Validity**

Next, we correlated the blink rate at 60% and 80% tracking ratios. The mean number of blinks per session was calculated from the trial level. We found a significant correlation between the blink rates at 60% and 80% tracking ratios (b = .98, p < .001), demonstrating good intra-validity.
